# The efficacy of cetuximab plus PD-1 inhibitors as salvage therapy in PD-1 refractory patients with recurrent or metastatic head and neck squamous cell carcinoma

**DOI:** 10.7150/jca.92689

**Published:** 2024-01-27

**Authors:** Rongrong Hui, Xiulan Liu, Zongyu Fan, Honghai Ji, Dongliang Wei, Guoxin Ren

**Affiliations:** 1 School of Stomatology, Weifang Medical University, Weifang, China.; 2 Department of Oral Maxillofacial-Head and Neck Oncology, Shanghai Ninth People's Hospital, Shanghai Jiao Tong University School of Medicine, Shanghai, China.; 3 Shanghai Key Laboratory of Stomatology & Shanghai Research Institute of Stomatology; National Clinical Research Center of Stomatology, Shanghai, China.; 4 National Clinical Research Center of Stomatology, Shanghai, China.

**Keywords:** programmed cell death protein 1 (PD-1) immunotherapy, cetuximab, salvage therapy, recurrent or metastatic head and neck squamous cell carcinoma (R/M HNSCC)

## Abstract

**Purpose:** The prognosis of patients with recurrent or metastatic head and neck squamous cell carcinoma (R/M HNSCC) that are refractory to programmed cell death protein 1 (PD-1) immunotherapy is relatively poor. The salvage therapy was rarely investigated and urgently needed.

**Methods:** We conducted a single center retrospective real-world study to explore the efficacy of cetuximab plus PD-1 inhibitors as salvage therapy in patients progressed from first-line immunotherapy.

**Results:** In the present study, 28 eligible patients were included between October 2020 and May 2023. By the cut-off date (Sep 24^th^, 2023), the objective response rate (ORR) was 46.4% (95% CI, 29.5%-64.2%). Kaplan-Meier survival analysis revealed the median progression free survival (mPFS) in the study was 6.87 months (95% CI, 4.77-8.97 months), and median overall survival (mOS) was 9.67 months (95% CI, 4.79-14.55 months). Multivariate Cox regression analysis indicated that ECOG performance status and best response to salvage therapy was found to be the prognosis factor of salvage therapy. For the safety, the most common treatment related adverse events (TRAEs) were rash (72.1%), anemia (64.3%) and fatigue (46.5%) during the salvage therapy. The most common potential irAEs were hypothyroidism (25%), and pneumonitis (14.3%). Only 3 patients (10.7%) experienced grade 3 TRAEs, and no treatment-related deaths occurred.

**Conclusions:** Our study showed the combination of cetuximab with PD-1 inhibitors might be a potential efficacy and safety choice in PD-1 refractory patients with R/M HNSCC which need further investigation.

## Introduction

Immunotherapy targeting programmed cell death protein 1 (PD-1) significantly improved the prognosis of patients with recurrent or metastatic head and neck squamous cell carcinoma (R/M HNSCC). In KEYNOTE-048 study, pembrolizumab combined with chemotherapy in total population or pembrolizumab alone in participants with PD-L1 combined positive score (CPS)≥1 obtained better survival benefit than EXTREME regimen (cetuximab plus cisplatin/5-fluorouracil) in the first-line treatment for patients with R/M HNSCC 1. Thus, anti-PD-1 immunotherapy gradually replaced traditional cetuximab-based regime or chemotherapy and is established as standard first-line treatment in R/M HNSCC 2.

50-60% patients with R/M HNSCC were primarily insensitive to the anti-PD-1 immunotherapy. With an 5-6 months median progression-free survival time 1, considerable amounts of patients with R/M HNSCC administrate second-line therapy due to secondary resistant to immunotherapy. In the current standard second-line treatment (single-agent chemotherapy or cetuximab or immunotherapy), the objective response rate (ORR) is reported to be 13%-14.6%, and the median progression free survival (PFS) time is approximately 2 months 3-5, indicating a relatively poor prognosis. The efficacy data are based on the patients relapsed from traditional cetuximab-based regime or cisplatin-based chemotherapy as first-line treatment. Scattered reports described the efficacy and prognosis of salvage therapy in patients with R/M HNSCC who relapsed from immune checkpoint inhibitor (ICI) therapy in the first-line treatment 6, however, few systematic studies have been investigated.

EGFR blocking initiate antibody-dependent cellular cytotoxicity (ADCC) and trigger enhanced adaptive and innate immune response 7-11. Therefore, cetuximab may work synergistically with ICI therapy and act as an effective salvage therapeutic agent refractory to immunotherapy theoretically. Here, we conducted a real-world study to investigate the efficacy and feasibility of cetuximab with PD-1 inhibitors salvage therapy in patients with R/M HNSCC that relapsed from anti-PD-1 immunotherapy.

## Materials and Methods

### Study design

This study was conducted in the Department of Oral and Maxillofacial-Head and Neck Oncology, the Shanghai Ninth People's Hospital affiliated to Shanghai Jiaotong University School of Medicine. We retrospectively reviewed the clinical data of patients diagnosed R/M HNSCC between October 2020 and May 2023. The inclusion criteria were patients with anti-PD-1 immunotherapy-containing therapy in the first-line treatment, tumor progression confirmed by radiological image after anti-PD-1 immunotherapy, administrated with at least one dose of cetuximab with PD-1 inhibitors therapy in the second-line treatment, without a history of cetuximab application in the first line treatment, without persistent immune-related AEs (irAEs, defined as adverse medical events potentially related to immunotherapy that occurs during immunotherapy) and relatively complete clinical and follow-up data were enrolled in the study. The exclusion criteria were the patients had any active autoimmune disease or a history of autoimmune disease; receiving immunosuppressive, or systemic hormonal therapy for immunosuppression; massive pleural fluid or ascites associated with clinical symptoms and requiring symptomatic management; active lung disease (interstitial pneumonitis, pneumonia, obstructive lung disease, asthma) or a history of active tuberculosis; having any uncontrolled clinical problems, including persistent or active (severe) infections, poorly controlled diabetes and heart disease (class III/IV congestive heart failure or heart block as defined by the New York Heart Association). Baseline characteristics, clinical history, efficacy and toxicity of treatment were collected from review of medical records. The study was approved by the Review Committee of the Shanghai Ninth People's Hospital, Shanghai Jiaotong University School of Medicine, and was conducted in accordance with the Declaration of Helsinki.

### Imaging analysis

Imaging analyses were conducted by computed tomography (CT), magnetic resonance imaging (MRI), which were interpreted by the radiologists every 2-4 dosing cycles. Tumor lesions were considered to have the longest diameter, and metastatic lymph nodes must have the shortest diameter of at least 15 mm. If symptoms developed during treatment, the patients were immediately examined and evaluated. Data were evaluated according to the Response Evaluation Criteria in Solid Tumors (RECIST) version 1.1.

### Outcomes

The primary endpoint was the best objective response rate (ORR), defined as the percentage of patients with 30% tumor shrinkage from baseline and was maintained over 4 weeks, and stable disease (SD) was defined as tumor volume ranged from 30% tumor shrinkage to 20% progression from baseline and maintained over 6 weeks according to the RECIST version 1.1. The secondary endpoints were progression free survival (PFS, defined as the time from treatment initiation to tumor progression or death due to any cause), overall survival (OS, defined as the time from treatment initiation to death due to any cause or patient review at cut-off time), and treatment related adverse events (TRAEs).

### Statistical analysis

The last follow-up for all included patients was Sep 24th, 2023. The ORR and 95% confidence interval (CI) were determined. PFS and OS were plotted using the Kaplan-Meier method, with the median and corresponding two-sided 95% CIs reported. Log-rank test was utilized for univariate analysis, and Cox regression model was performed for multivariate analysis. Statistical analysis was performed using SPSS software (version 26.0; IBM Corp., Armonk, NY, USA).

## Results

### Baseline characteristics of patients

**Figure 1:**
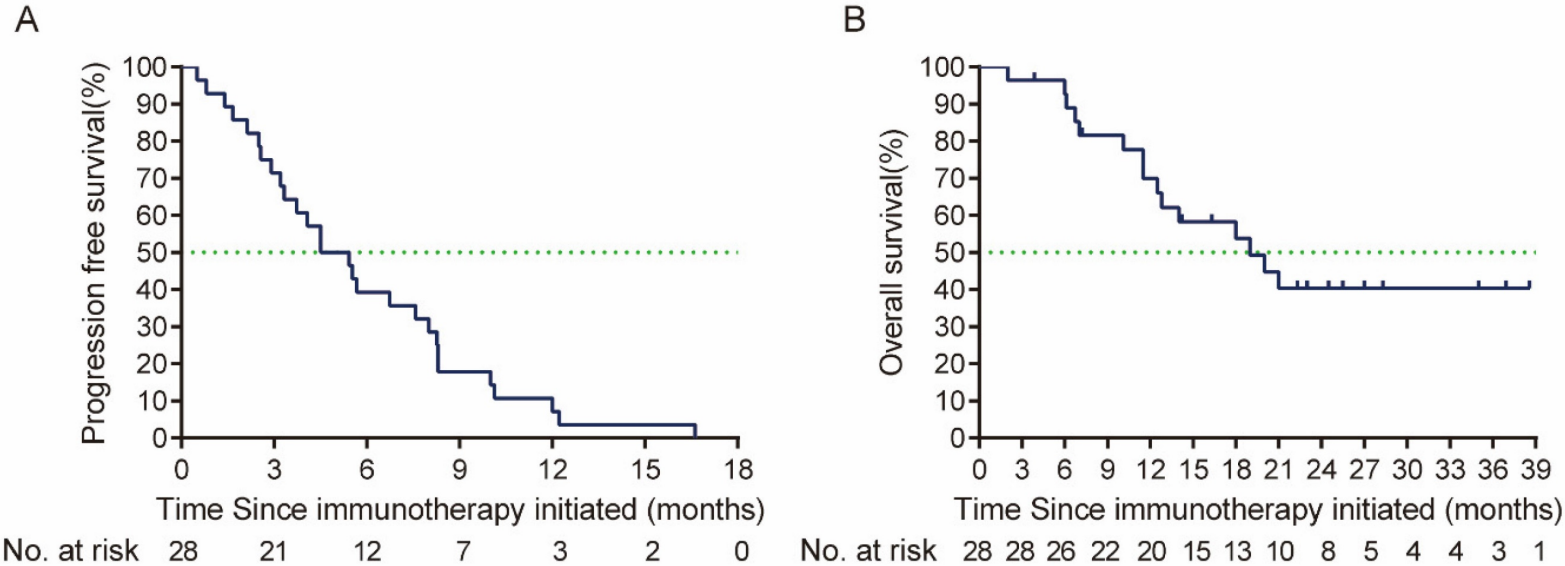
Kaplan-Meier curve of (A) PFS (defined as the time from the first line treatment initiation to tumor progression or death due to any cause) and (B) OS (defined as time from the first line treatment initiation to death due to any cause or patient review at cut-off time) of PD-1 immunotherapy in the first line treatment. PFS: Progression free-survival; OS: overall survival.

From October 2020 to May 2023, 28 patients with R/M HNSCC that received anti-PD-1 immunotherapy in the first-line treatment were finally included. The baseline characteristics of patients were presented in **Table 1**. Prior to the first line treatment, 19 (67.9%) patients were only local or regional recurrent, 6 (21.4%) had both recurrent and metastatic lesions, and 3 (10.7%) had metastatic lesions alone. Among the enrolled patients, 25 (89.3%) patients were oral squamous cell carcinoma (OSCC), and 3 (10.7%) patients were oropharyngeal cancer. Twenty-two (78.6%) patients received PD-1 immunotherapy with chemotherapy, 4 (14.3%) patients received PD-1 immunotherapy with VEGFR inhibitor (apatinib), and 2 patient (7.1%) received PD-1 immunotherapy alone in the first line treatment. One patient achieved CR when receiving PD-1 first-line treatment. However, the tumor relapsed after 21 times of PD-1 inhibitor administration, and then the patient received PD-1 with cetuximab second-line treatment. Overall, the ORR to PD-1 immunotherapy in the first line treatment was 50% (95% CI, 32.6%-67.4%) in total population, median PFS was 4.5 months (95% CI, 2.61-6.39 months), and median OS was 19.0 months (95% CI, 10.23-27.77 months) **(Figure 1)**.

### The efficacy of cetuximab with PD-1 inhibitors salvage therapy

**Figure 2:**
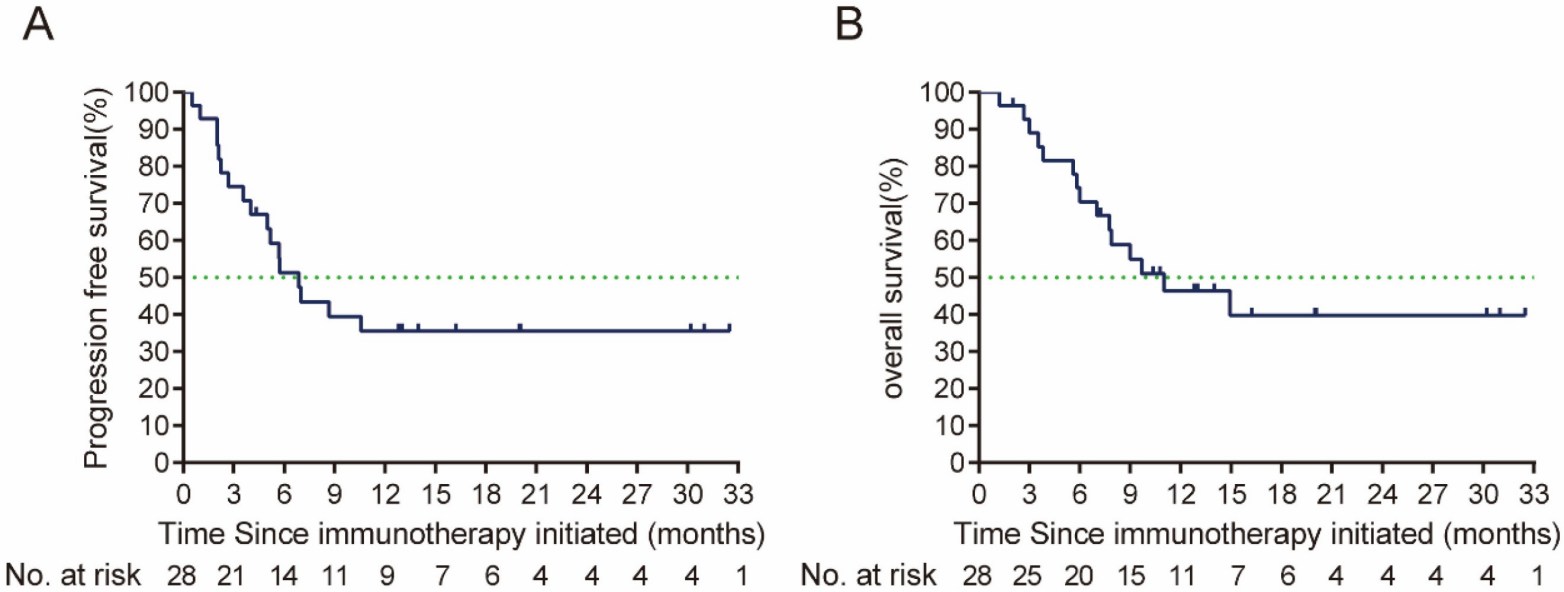
(A) PFS (the time from the salvage treatment initiation to tumor progression or death due to any cause), and (B) OS (time from the salvage treatment initiation to death due to any cause or patient review at cut-off time) from salvage therapy after ICI failure. PFS: Progression free-survival; OS: overall survival; ICI: immune checkpoint inhibitors.

By the cut-off time (Sep 24^th^, 2023), 13 (46.4%) patients were still alive. Prior to the second line treatment, 1 patient who were only local or regional recurrent had metastatic lesions. Six patients had a ≥2 ECOG performance status **(Table 2)**. In the cetuximab plus PD-1 inhibitors salvage therapy, the ORR was 46.4% (95% CI, 29.5%-64.2%), disease control rate (DCR) was 82.1%. In the survival analysis, with a median follow up time of 9.33 months (ranged from 1.2 to 32.5 months), the median PFS in the study was 6.87 months (95% CI, 4.77-8.97 months), and median OS was 9.67 months (95% CI, 4.79-14.55 months) **(Figure 2)**. By univariate analysis, eastern Cooperative Oncology Group (ECOG) performance status and primary tumor site were associated with the ORR of salvage therapy, ECOG performance status and best response to salvage treatment were associated with median PFS and median OS of salvage therapy **(Table 3, 4)**. By multivariate analysis, ECOG performance status was found to be the prognosis factor of PFS and OS of salvage therapy, and best response to salvage therapy was found to be the prognosis factor of PFS of salvage therapy **(Table 5)**.

### The toxicity of cetuximab with PD-1 immunotherapy salvage therapy

Most of patients (23/28, 82.1%) experienced at least one TRAE, and no treatment-related deaths occurred. The most common TRAEs were rash (72.1%), anemia (64.3%) and fatigue (46.5%). None of patients had persistent irAE prior to the salvage therapy. In the salvage therapy, nine (32.1%) patients experiencedirAEs. The most common potential irAEs were hypothyroidism (25%), and pneumonitis (14.3%, **Table 6)**.

Only 3 patients (10.7%) experienced grade 3 TRAEs, one patient (3.6%) experienced hypothyroidism, one patient (3.6%) experienced pneumonitis, and one patient (3.6%) experienced rash. The patients experienced grade 3 pneumonitis recovered by hormone therapy (methylprednisolone 2mg/kg/day) within 10 days, but the patient discontinued salvage therapy permanently due to toxicities. The other two patients continued the treatment when the grade 3 TRAEs recovered **(Table 6)**.

## Discussion

Our retrospective real-world study firstly reported the efficacy and safety of cetuximab with PD-1 immunotherapy salvage therapy in patients with R/M HNSCC that were refractory to the PD-1 immunotherapy. Our results indicated a potentially improved ORR, median PFS and OS compared with the results in the second-line treatment of R/M HNSCC patients reported by previous studies 3-5.

EGFR blocking reduce tumor cell proliferation, survival, angiogenesis and migration by inhibiting the activity of MAPK and PI3K signaling. Cetuximab could reverse the immune suppressive effect by increasing the activity of cytotoxic lymphocytes (CTLs), decreasing the number and activity of Tregs, and increasing the expression of MHC I and MHC II 12-14. Therefore, PD-1 immunotherapy and cetuximab have synergistic effect theoretically. Sacco AG et al reported that the efficacy and safety of pembrolizumab plus cetuximab in patients with R/M HNSCC who with no previous PD-1, PD-L1, or EGFR inhibition after recurrent or metastatic. It revealed a 45% overall response rate by 6 months (15 of 33 participants) with a significant survival benefit 15. Another study investigated the efficacy of pembrolizumab combined with afatinib, a type of EGFR-TKI. The study also showed the favorite response and survival in platinum-refractory R/M HNSCC patients 16. These studies indicated a promising clinical activity of PD-1 immunotherapy combined with EGFR blocking in patients with R/M HNSCC. The prognosis of patients with R/M HNSCC by current standard second-line therapy was relatively poor (the average ORR was 14% and median PFS was 2 months). However, the efficacy of salvage therapy in patients who refractory to PD-1 immunotherapy was rarely reported. Saleh K et al reported the ORR of salvage chemotherapy in patients who refractory to PD-1 immunotherapy was 30%, median PFS was 3.6 months and median OS was 7.8 months respectively 17. In our retrospective study, we observed that a potentially improved ORR and survival benefit in cetuximab with PD-1 immunotherapy salvage therapy compared with previously reported (ORR ranged 30-42%, mPFS ranged 3.6-4.2 months, and mOS ranged 7.8-8.4 months) 18, 19.

Previous study reported the response to ICI therapy might enhance the efficacy of salvage chemotherapy in R/M HNSCC patients 17. Our results observed better response to first line ICI therapy tend to be more sensitive to salvage therapy, but significant difference was not reached. These results might owe to the relatively small sample size and limited follow up time and deserve further confirmation. ECOG performance status was reported to be associated with higher response and prolonged OS in HNSCC patients by the cetuximab-containing therapy 20-21. ECOG performance status affected the response and prognosis of salvage therapy on R/M HNSCC patients in our study, and patients with only local or reginal recurrence had a prolonged median OS. Oropharyngeal cancer was reported to be more sensitive to EGFR-target therapy and immunotherapy than oral cancer 22, 23. Our observations also found that oropharyngeal cancer had a better ORR of salvage therapy by univariate analysis, suggesting that the combination of cetuximab and PD-1 inhibitors might be more effective for patients with oropharyngeal cancer.

The tolerance to treatment is always poor when the patients progressed from first line therapy, and the toxicity of chemotherapy-containing salvage therapy is relatively high in the previous studies 24, 25. Cabezas-Camarero S et al^.^ retrospectively analyzed the toxicity of 23 patients who received cetuximab-based combinations after progression on ICI therapy. It revealed that 100% patients experienced grade 1 or 2 AEs, 65% patients experienced grade 3 or worse AEs 26. Suzuki S et al also reported that grade 3 or worse hematologic toxicities were occurred in 22.3% patients and grade 3 or worse non-hematologic toxicities were occurred in 16.8% patients who received cetuximab with paclitaxel after progression following ICI therapy 27. In our study, we observed a significantly lower occurrence of grade 3 or worse AEs by the combination of cetuximab and PD-1 inhibitors than previously reported, indicating that cetuximab with PD-1 inhibitors is well tolerable as salvage therapy.

Our study still had several limitations including that our study was a single center retrospective investigation and relative reduced sample size. The follow up time of enrolled patients also need to be extended. In addition, the regime of first-line PD-1 immunotherapy is heterogeneous and may interfere with the subsequent analysis. Hence, further investigations are needed to optimize R/M HNSCC pharmacotherapy.

In conclusion, our study showed an increased response rate and improved survival of cetuximab-containing salvage therapy in patients with R/M HNSCC after progression on PD-1 immunotherapy in the first-line treatment. The results suggested that early progression after first-line PD-1 immunotherapy might indicate better response to cetuximab target therapy. A larger scale randomized, double-blind prospective trials are required to further confirm the deduction and optimize the treatment of R/M HNSCC.

## Figures and Tables

**Table 1:** Baseline characteristics of patients prior to first-line treatment

Characteristics	Participants
Median age, years (range)	58 (40‐83)
Sex	
Female	9 (32.1%)
Male	19 (67.9%)
ECOG performance status	
0	2 (7.1%)
1	26 (92.9%)
Smoking history	
Never	14 (50.0%)
Former	13 (46.4%)
Current	1 (3.6%)
Alcohol use	
Never	13 (46.4%)
Former	15 (53.6%)
Current	0 (0%)
Primary tumor site	
Oral cavity	25 (89.3%)
Oropharynx	3 (10.7%)
Recurrence pattern	
Local or reginal recurrence only	19 (67.9%)
Local or reginal recurrence and distant metastases	6 (21.4%)
Distant metastases only	3 (10.7%)
HPV infection	
Positive	2 (7.1%)
Negative	26 (92.9%)
First-line treatment	
ICI therapy with chemotherapy	22 (78.6%)
ICI therapy with anti-VEGFR target therapy	4 (14.3%)
ICI therapy alone	2 (7.1%)
Best response to first-line treatment	
CR	1(3.6%)
PR	13 (46.4%)
SD	7 (25.0%)
PD	7 (25.0%)

ICI: immune checkpoint inhibitor; ECOG: Eastern Cooperative Oncology Group; CR: complete response, PR: partial response, SD: stable disease, PD: progressive disease.

**Table 2:** The characteristics of patients prior to the salvage therapy

Characteristics	Participants
Median age, years (range)	60 (40‐84)
ECOG performance status	
0	0 (0%)
1	22 (78.6%)
2-3	6 (21.4%)
Recurrence pattern	
Local or reginal recurrence only	18 (64.3%)
Local or reginal recurrence and distant metastases	7 (25.0%)
Distant metastases only	3 (10.7%)

ECOG: Eastern Cooperative Oncology Group.

**Table 3:** The univariate analysis of ORR of salvage therapy

Characteristics	Patients with PD or SD	Patients with PR or CR	P value
Median age, years			
< 60 years	5 (17.9%)	7 (25.0%)	0.274
≥60 years	10 (35.7%)	6 (21.4%)	
ECOG performance status			
0-1	9 (32.1%)	13 (46.4%)	0.010
2-3	6 (21.4%)	0 (0%)	
Sex			
Female	7 (25.0%)	2 (7.1%)	0.077
Male	8 (31.8%)	11 (36.4%)	
Primary tumor site			
Oral cavity	15 (53.6%)	10 (35.7%)	0.049
Oropharynx	0 (0%)	3 (10.7%)	
Recurrence pattern			
Local or reginal recurrence only	9 (32.1%)	9 (32.1%)	0.221
Local or reginal recurrence and distant metastases	3 (10.7%)	4 14.3%)	
Distant metastases only	3 (10.7%)	0 (0%)	
Best response to first-line treatment			
CR+PR	5 (17.9%)	9 (32.1%)	0.058
SD+PD	10 (35.7%)	4 (14.3%)	
Type of therapy in first-line treatment			
ICI therapy with chemotherapy	13(46.4%)	9(32.1%)	0.262
ICI therapy without chemotherapy	2(7.1%)	4(14.3%)	

ORR: objective response rate; ICI: immune checkpoint inhibitor; ECOG: Eastern Cooperative Oncology Group; CR: complete response, PR: partial response, SD: stable disease, PD: progressive disease.

**Table 4:** The univariate analysis of PFS and OS of salvage therapy

Characteristics	Median PFS	P value	Median OS	P value
Median age, years				
< 60 years	7.0 (0.00-14.78)	0.744	11 (3.09-18.91)	0.911
≥ 60 years	5.7 (2.32-9.08)		9 (5.57-12.43)	
ECOG performance status				
0-1	10.6	<0.001	14.9	<0.001
2-3	2.0 (0.87-3.13)		3.5 (2.57-4.49)	
Sex				
Female	5.2 (3.68-6.67)	0.497	9.0 (5.18-12.83)	0.622
Male	8.7 (2.63-14.71)		unreached	
Recurrence pattern				
Local or reginal recurrence only	unreached	0.122	unreached	0.077
Local or reginal recurrence and distant metastases	5.7 (4.34-7.06)		7.9 (5.64-10.10)	
Distant metastases only	5.0 (0.36-9.64)		7.8 (0.99-14.56)	
Best response to first-line treatment				
CR+PR	6.9 (4.54-9.19)	0.280	unreached	0.078
SD+PD	5.2 (0.00-14.17)		5.6 (0.00-15.53)	
Type of therapy in first-line treatment				
ICI therapy with chemotherapy	5.7 (2.92-8.48)	0.568	9.0(4.53-13.47)	0.354
ICI therapy without chemotherapy	7.0 (4.35-9.65)		unreached	
Best response to salvage treatment				
CR+PR	unreached	<0.001	unreached	0.001
SD+PD	3.8 (1.19-5.95)		5.8(3.05-8.61)	

PFS: progression free survival; OS: overall survival; ICI: immune checkpoint inhibitor; ECOG: Eastern Cooperative Oncology Group; CR: complete response, PR: partial response, SD: stable disease, PD: progressive disease.

**Table 5:** The multivariate analysis of PFS and OS of salvage therapy

Characteristics	HR	95% HR CI	P value
PFS			
ECOG performance status			
0-1	1		0.005
2-3	0.064	0.009-0.437	
Best response to salvage treatment			
CR+PR	1		0.031
SD+PD	2.002	0.533-7.516	
OS			
ECOG performance status			
0-1	1		0.015
2-3	0.099	0.015-0.641	

PFS: progression free survival; OS: overall survival; ECOG: Eastern Cooperative Oncology Group; HR: hazard ratio; CI: confidence interval.

**Table 6:** The toxicity of cetuximab with PD-1 immunotherapy

Treatment-related adverse events	Grade 1	Grade 2	Grade 3	Grade 4	Grade 5
Hypothyroidism	4(14.3%)	2(7.1%)	1(3.6%)	0(0%)	0(0%)
Pneumonitis	2(7.1%)	1(3.6%)	1(3.6%)	0(0%)	0(0%)
Rash	16(57.1%)	6(21.4%)	1(3.6%)	0(0%)	0(0%)
Fatigue	8(28.6%)	5(17.9%)	0(0%)	0(0%)	0(0%)
Anemia	13(46.4%)	5(17.9%)	0(0%)	0(0%)	0(0%)
